# The 1001 nights-cohort – paving the way for future research on working hours, night work, circadian disruption, sleep, and health

**DOI:** 10.1007/s10654-025-01201-4

**Published:** 2025-02-07

**Authors:** Kirsten Nabe-Nielsen, Anne Emily Saunte Fiehn Arup, Mette Sallerup, Rikke Harmsen, Anna Sofie Ginty, Marie Tolver Nielsen, Anne-Sofie Rosenfeldt Jensen, Anders Aagaard, Vivi Schlünssen, Ann Dyreborg Larsen, Anne Helene Garde

**Affiliations:** 1 The National Research Centre for the Working Environment, Copenhagen, Denmark; 2 Department of Public Health, University of Copenhagen, Copenhagen, Denmark; 3 Department of Public Health, Research Unit for Environment, Occupation and Health, Danish Ramazzini Centre, Aarhus University, Aarhus, Denmark

**Keywords:** Wearables, Working hours, Cancer, Cardiometabolic diseases, Mental health, Biobank

## Abstract

Night work and circadian disruption are linked to major public health challenges, e.g. cancer, cardiometabolic disease, and accidents. We established the *1001 nights-cohort* to explore mechanisms underlying health effects of night work and circadian disruption. 1075 female hospital employees participated from September 2022 to April 2024. The data collection included a questionnaire, a blood sample, anthropometric measures, and sleep actigraphy and sleep diaries across 14 days. In subsamples, light exposure, physical activity, skin temperature, and blood glucose were measured continuously for 7 days, and saliva samples were collected five times across one day. The cohort consists of 2- and 3-shift workers with night work (66%), permanent night workers (7%), permanent evening workers or 2-shift workers without night work (9%), and permanent day workers (18%). Data comprise 4553 day shifts, 997 evening shifts, 1963 night shifts, and 6458 days without work. The poorest health was observed among permanent night workers and the group of shift workers *without* night work. The 1001 nights-cohort is the most comprehensive data within night work and working hour research due to the combination of questionnaires, biomarkers, technical measurements, and possibilities for linkage to historical and future register-based information about working hours from the Danish Working Hour Database (DAD) and diagnoses. With its repeated measurements within the same individual, the cohort will advance research on physiological and behavioral mechanisms underlying health effects of working hours, night work, and circadian disruption and deliver important scientific input for updating guidelines on healthy scheduling of working hours.

## Background

Globally, occupational exposures play a significant role for public health: Among the annually registered 2.9 million work-related deaths, the most frequent causes were circulatory diseases, cancer, respiratory diseases, and occupational injuries [1]. These deaths and the 180 million disability adjusted life-years attributed to work may be preventable. Additionally, as many hazardous occupational exposures are socially skewed, they also have implications for social inequality in health [2].

Night work is an occupational exposure with a potential major impact on public health: It is acknowledged by the International Agency for Research on Cancer as probably carcinogenic in relation to breast, prostate, colon and rectum cancer [3]. Furthermore, night work is also prospectively associated with cardiovascular and metabolic risk factors and diseases [4, 5]. Other working hour characteristics also have implications for health: Long weekly working hours are suggested as the single occupational risk factor with the largest number of attributable deaths [6], and short recovery time between shifts (i.e. quick returns) has been linked with a higher risk of accidents [7, 8].

In Europe, 24% of the population work night shifts [9], and night work is unavoidable in many jobs, e.g. within healthcare, police, production, and transport. Thus, there is a potential for improving health through interventions targeting the organization of, e.g. night shifts, duration of shifts, and recovery periods between shifts. Such interventions may reduce associated health risks, e.g. through improved sleep [10, 11].For this reason, further knowledge about how to organize working hours in general, and night work in particular, is essential [12].

Night work is suggested to enact its effect on health-related outcomes through physiological, psychological and behavioral mechanisms [13]. Circadian disruption and sleep deprivation appear to play a particularly important role [13]. Importantly, exposure to night work can serve as a feasible model of circadian disruption in humans with a potential to advance research in several research fields.

More knowledge about mechanisms linking night work with health outcomes can help to disentangle causal effects from non-causal associations and direct attention towards potential interventions. The overall purpose of the 1001 nights-cohort is, therefore, to enable the investigation of mechanisms underlying health effects of working hour characteristics, particularly night work and the consequent circadian disruption. With its vast amount of data and possibilities for linkage to historical and future register data, the potential for use of the cohort for evolving research topics within the research area of night work and circadian disruption is almost unlimited. The objective of the current paper, is to present a profile of the participants of the 1001 nights-cohort, including (i) a description of the characteristics of participants across work schedules (e.g. with and without night work), (ii) a presentation of the exposure to night shifts among both permanent night workers and 2- or 3-shift workers with night work, and (iii) a comparison of the cohort with the source population.

## Study design and methodology

### Study design, data collection and data sources

Data for this cohort study were collected from September 2022 to April 2024 in the five Regions of Denmark. The regions are administrative entities responsible for the public secondary health care system, i.e. psychiatric and somatic hospitals. In Denmark, health care coverage is universal and hospitals are mainly public.

**Fig. 1:**
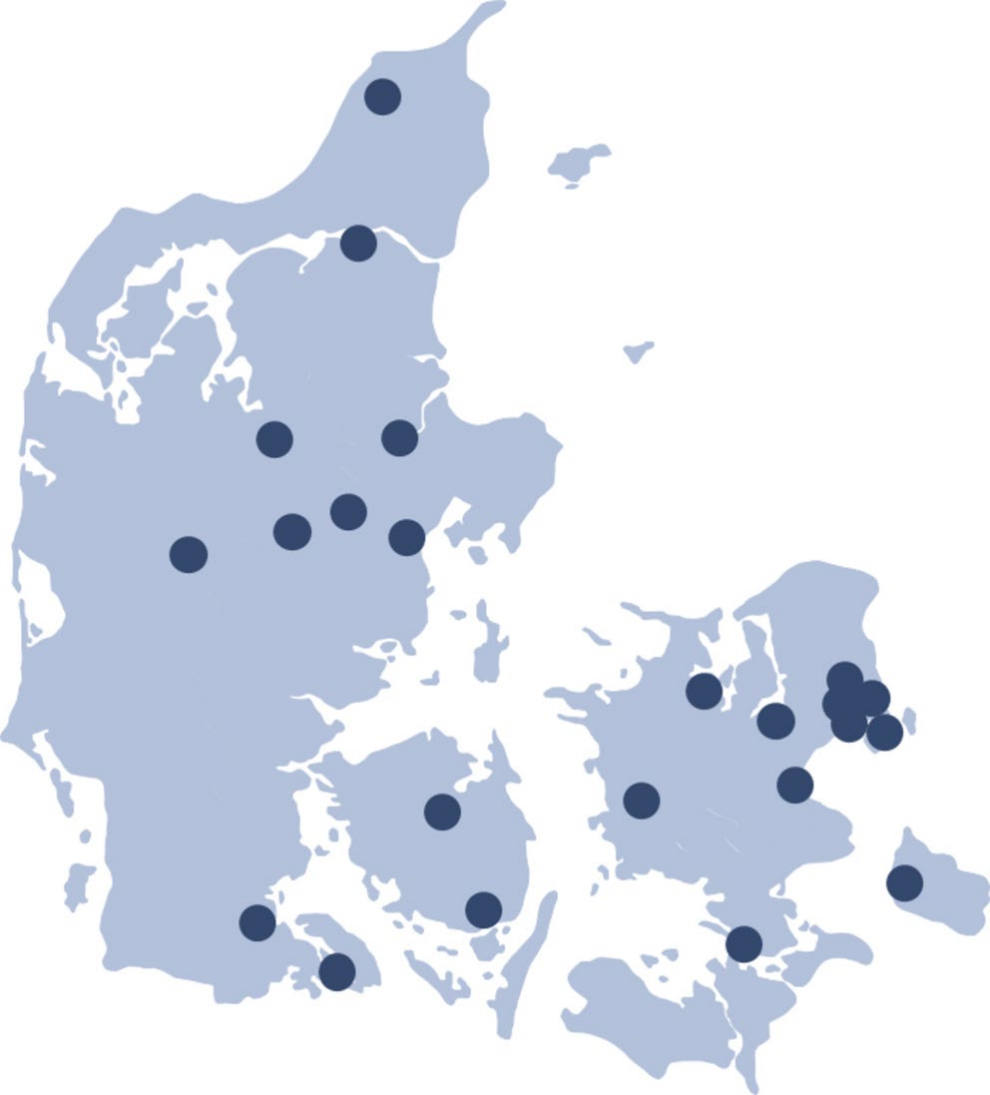
Overview of geographical location of the data collection in Denmark

As part of the recruitment and data collection, the research team visited 26 sites (hospitals) across the country (Fig. 1). Each site was visited for 1–4 days. A smaller group of participants visited the research team at The National Research Centre for the Working Environment (*Danish*: Det Nationale Forskningscenter for Arbejdsmiljø, NFA), Copenhagen, Denmark.

The baseline data collection consisted of data from the examination day (“day zero”), blood samples and a background questionnaire. Hereafter, all participants were followed for 14 consecutive days via sleep diaries and objective measurements of sleep with wrist actigraphy. During the first 7 days after the baseline data collection, subsamples were also followed with objective, continuous measurements of light exposure, physical activity, skin temperature, and blood glucose. Saliva samples were collected across one day in a subsample.

### Inclusion and exclusion criteria

Women working in one of the five Danish regions were eligible for inclusion in the cohort. The study population was delineated to female employees for three reasons: (i) The cohort supplements existing studies of night work among men; (ii) women are dominant in the health care sector; (iii) hormones and biological markers differ between sexes necessitating sex stratified analyses and hence, a larger study population.

Employees could participate regardless of their work schedule as long as they worked a minimum of 28 h a week. The ambition was that the cohort should consist of 70% night shift workers, 10% permanent night workers, and 20% day or evening workers. Pregnant women or women trying to conceive in the 14 days period were not eligible for inclusion due to potential changes in hormone levels and other biomarkers measured in blood and saliva samples. Otherwise, we did not apply any health-related inclusion or exclusion criteria for the cohort (see Table 1 for further details about inclusion criteria for data collection in subsamples).

### Recruitment of hospitals and participants

Participants were recruited through the workplaces (i.e. hospitals). Prior approval from each of the five Regions’ workplace cooperation committees (*Danish*: MED-udvalg) consisting of representatives for management and employees was obtained. A contact persons in each region communicated information about the project to the management of the hospitals in each region. Decisions about participation at the hospital level was then taken either by management alone or in collaboration between employers’ and employees’ representatives. We did not systematically collect any information about reasons for participation or non-participation at the organizational level, yet, lack of resources was at play. Some hospitals, which did not initially sign up for the project, signed up at a later time as resources became available. The role of the hospitals in the recruitment process was to assign a local contact person, distribute information about the project (received from the research group), and to book rooms for the baseline examination of the participants.

To obtain awareness about the research project, information was communicated through the hospitals’ own communication channels, at the project’s website, via social media and by contact to labor unions. If possible, flyers were distributed at the hospitals (e.g. in the employees’ canteen) a week before or during the data collection.

Recruitment was done at an individual level: Each participant booked a time-slot for participation, typically starting between 7:00 am and 16:30 pm. These “opening hours” allowed employees to participate regardless of their shift type. Enrollment, examination and blood sampling took 30–45 min. Participation could take place during working hours (if approved by the management) or during leisure-time.

### Participants

A total of 1075 individuals signed the consent of participation statement and provided data for the study. Participants were nurses, nursing assistants, midwifes, medical doctors, biomedical laboratory scientist, and administrative staff employed either at a somatic or psychiatric hospital. The participants worked in a broad range of departments, e.g. medical, surgical, emergency or outpatient wards. The participants represented various medical specialties, e.g. psychiatry, orthopedic surgery, gynecology and obstetrics, radiology, and oncology.

Table 1 presents an overview of the data sources, the inclusion criteria for each type of data collection, and number of participants in the subsamples contributing with data for each specific data source. An overview of the number of participants with combinations of available data can be found at the project’s webpage (www.nfa.dk/1001nights).

**Table 1:** Overview of data sources and inclusion criteria for the specific parts of the data collection according to the protocol. The examination, collection of blood sample, and distribution of the background questionnaire were taking place on the day of enrollment

Data source	Information	Time of collection/ wearing time	*N* (individuals)
Examination ^A^	Background information, blood pressure, anthropometrics	Once at baseline	1075
Self-reported background questionnaire ^A^	Sociodemographic factors, sleep habits, medication, health, chronotype	Once at baseline	994
Self-reported sleep diaries ^A^	Timing of work, sleep, and meals, symptoms	Once every day for 14 days	1061
Blood sample ^A^	For example, cholesterol, glycated hemoglobin, hs-CRP, and telomere length	Once at baseline	1031
Saliva samples ^A, B/C^	For example, cortisol and melatonin	5 samples at specific time points during one 24-h day	286
Wrist actigraphy (Actigraph) ^A^	Sleep, physical activity	Continuous, 14 days	999
Accelerometers on thigh and back (SENS) ^A,C/D^	Sleep, physical activity, skin temperature	Continuous, 7 days	698
Light exposure device (HOBO) ^A, B/C^	Light, ambient temperature	Continuous, 7 days	307
Interstitial blood glucose (DEXCOM) ^A, C^	Continuous blood glucose	Continuous, 7 days	51

(A) All participants, i.e. female employees employed in a Danish region (the hospital sector). (B) Either at least two consecutive night shifts during the first seven days after enrollment, two consecutive night shifts during the days leading up to the enrollment, or participants with permanent day work and at least two consecutive work days leading up to or during the first seven days after enrollment. (C) At least one day shift and at least one night shift during the first seven days after enrollment, non-diabetic, and not having an MR-scan during the study period.D) At least four workdays during the first 7 days after enrollment. *NB The inclusion criteria were not mutually exclusive*

### Ethics and adherence to general data protection regulation

The study is approved by the Danish National Committee on Health Research Ethics (H-21077744) and follows the regulations of the Danish Data Protection Agency including an internal registration and risk assessment.

On the examination day, the participants received written and oral information about the project, including potential discomfort and complications related to participation, and they signed an informed consent form. Participation was voluntary and participants could withdraw from the project at any time. We emphasized that employers were not informed about who participated in the project and health information from individual participants was treated confidentially.

Individual feedback was given in the case of a high depression score or abnormal levels of blood lipids and glycated hemoglobin. Furthermore, all participants with available data received an individual sleep report (sleep diary data) and a glucose report (continuous glucose measurement (CGM) data).

### Measurements on the examination day

#### Blood pressure

Blood pressure was measured three times with an Omron 3 Comfort blood pressure monitor on the upper right arm after the participants had rested in a sitting position for approximately 15 min (while receiving information about the project). Participants were instructed to sit in an upright position with both feet on the floor and asked not to talk during the measurement. The mean of the two last measurements was calculated and recorded.

#### Height and weight

The participants were instructed to be barefoot, empty their pockets and take off outerwear such as jackets and thick sweaters. Height was measured up against a wall using a Seca 217 altimeter measuring to nearest 0.1 cm. For weight measurements, we used an electronic Seca 803 Scale measuring to nearest 0.1 kg.

#### Hip and waist circumference

Hip and waist circumferences were measured with a regular measurement band. Waist was measured between the lower rib margin and the hip approximately at the level of the belly button, while hip circumference was measured where the participants were broadest. Participants were asked to empty their pockets and take of jackets and thick sweaters. Measurements were registered in centimeters and rounded to nearest 0.5 cm.

#### Blood sample

Up to 50 ml of blood was collected from each participant from the antecubital vein by venipuncture using Vacutainer tubes. The participants were non-fasting prior to sampling. Information on time of sampling, alcohol use and physical activity up to four hours before sampling was recorded. 2 ml of blood from each participant was kept as whole blood. After 15 min at room temperature, the rest of the blood was centrifuged for 15 min at 4000 rpm most often within 60–90 min after sampling. Afterwards, the samples were separated into 1 ml aliquots of serum, EDTA plasma, and cell fractions and stored at -80 ºC at NFA.

### Background questionnaire

The background questionnaire was electronically distributed via the participants’ email. Participants were encouraged to complete the background questionnaire as soon as possible; no reminders were sent. The questionnaire contained 57 questions about sociodemographic factors, working hours and working hour preferences, sleep, health behaviors, health, medication use and COVID-19. A comprehensive Morningness-Eveningness Questionnaire (MEQ) was applied, and a MEQ-score was calculated according to the guidelines [14]. Likewise, we used the Major Depression Inventory (MDI) and calculated a scale score according to the guidelines [15]. Sleep questions were inspired by Karolinska Sleepiness Questionnaire [16]. A full overview of the questionnaire is available at the project’s webpage (www.nfa.dk/1001nights).

### Sleep diaries

**Fig. 2:**
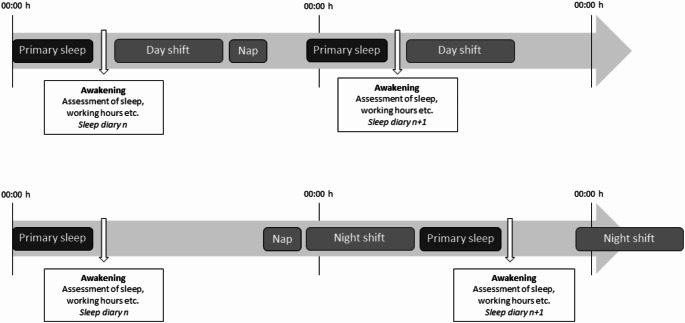
Example of timing of collection of diary data after a day and a night shift. Participants were instructed to fill out the sleep diary after their primary sleep covering all activities across the 24 h day (sleep diary 1) or since last sleep diary was filled out (sleep diary 2–14)

Sleep diaries were distributed via the participants’ email every morning at 5:00 for 14 days, and the participants were instructed to fill out the sleep diary immediately after termination of their primary sleep (Fig. 2). The sleep diary covered timing of working hours, sleep, physical activity, meals, and snacks, sleep quality, psychosocial and physical job demands, and symptoms since the previous primary sleep. The goal was to cover all activities across the last 24 h (sleep diary 1) or since the last sleep diary was filled out (sleep diary 2–14). A full overview of the sleep diaries is available at the project’s webpage (www.nfa.dk/1001nights). In total, 13,768 sleep diaries were completed (hereof 40 partially completed) yielding a response rate of 92.5%; 803 participants completed all 14 sleep diaries.

### Coding of working hours

Based on information on shift start and shift end reported in the sleep diaries, the participants’ working hours were categorized into shift types. Shift types were classified as “day shift” (≥ 3 h between > 06:00 and < 21:00); “evening shift” (≥ 3 h between ≥ 18:00 and < 02:00); and “night shift” (≥ 3 h between ≥ 23:00 and ≤ 06:00) [17]. The definitions were not mutually exclusive, and the categorization was prioritized in the following order: night > evening > day. Shifts with a duration of less than 3 h were not categorized. Some participants worked two shifts within a 24 h day, e.g., a day shift followed by a night shift. Days off included all days without work, including recovery days (i.e. days without work after a night shift), vacation, and sick leave.

During the study period, the 13,768 sleep diaries, provided us with information about 4553 day shifts, 997 evening shifts, 1963 night shifts, 77 short shifts (< 3 h), 14 work periods that could not be categorized due to missing information, and 6458 days without work; 294 days had two periods of work registered (i.e. double shifts).

### Saliva samples

In total, 286 participants, stemming from two different subsamples, collected five saliva samples across one 24-h day. Saliva sampling was conducted when waking up (before brushing teeth), 45 min after awakening, 4 h after awakening, 10 h after awakening and right before going to bed (before brushing teeth). The participants were advised not to change their daily routines during the sampling and were instructed not to collect the 10-h sample, if they had already gone to bed.

The first subsample (*n* = 235, inclusion criteria B, Table 1) were instructed to tip a cotton tampon into the mouth without touching it with the fingers, chew it for approximately two minutes, and then spit it back into the tube. Two tubes (Salivette^®^, one cotton swab and one synthetic swab, both neutral, SARSTEDT) were used at each measurement point. The second subsample (*n* = 51, inclusion criteria C, Table 1) were instructed to collect saliva samples by salivating directly into a single tube (conical tube, without swab, SARSTEDT), ensuring that there was at least 1 ml of clear saliva in each tube for all five collection times.

All the participants were asked to note the specific sampling time on a label on the saliva tube and to fill out a log book answering four questions regarding their intake of coffee and cigarette smoking within the past four and past two hours, respectively, leading up to the sampling. Participants were instructed to store the samples in a refrigerator or freezer until all samples were collected and returned via postal mail together with the log book. When received at NFA, saliva samples were stored at -80˚ Celsius. All log books were digitalized and validated by two independent researchers.

### Technical measurements of sleep (wrist actigraphy)

999 participants wore an accelerometer (Actigraph xGT3x-BT) for 14 consecutive days following the examination day. The Actigraphs were set up with a sampling rate of 30 Hz, and data were averaged across epochs of 60 s. The participants were instructed to wear the Actigraph on the wrist of their non-dominant hand for as much of the 24-h day as possible and most importantly during sleep. For hygienic reasons most participants took off the Actigraph during working hours as well as when showering.

### Technical measurements of physical activity (accelerometers on thigh and back)

We received accelerometer (SENS Motion) data from 698 participants, who wore them for seven consecutive days. The participants had one accelerometer placed in the middle of the upper back and one placed in the middle of the right thigh patched by a researcher on the examination day. The accelerometer had a sample rate of 12.5–25 Hz (depending on the device) allowing physical activities such as walking, running, sitting and forward bending to be estimated. The algorithms are a further development of the algorithms described by Skotte et al. [18]. The SENS device also measured skin temperature.

### Light exposure measurements

307 participants wore a device measuring light exposure (HOBO Pendant^®^ Temperature/Light Data Logger) for seven consecutive days following the examination day. The device had a sampling rate of 1 per 120 s. The device was attached to the participants clothing with a clip and participants were instructed to attach it by clipping it on the outermost layer of clothing in shoulder/collar height to approximate retinal light exposure. The participants were instructed to wear the device as much as possible during the examination period including both work and leisure time. When sleeping the device should be placed next to the bed with the measuring side facing up. If the participants did not wear the device for a longer period of time (e.g. forgetting to wear it for more than 15 min), they were instructed to note it in a log book. Upon return, all log books were digitalized and validated by two independent researchers.

### Interstitial glucose measurements

51 participants wore a glucose sensor (DEXCOM G6), measuring glucose levels in the interstitial fluid for seven consecutive days following the examination day. The DEXCOM G6 system records the glucose level every 5 min. The sensor was inserted underneath the skin surface on the abdomen or upper arm by trained staff and attached to the participant’s skin using adhesive tape. Data were transmitted to a receiver from the sensor. The receiver should be within 6 m of the sensor to allow transmission and recording of data. The participants were instructed to adhere to their usual daily habits, especially timing and content of meals, while wearing the device. Data collection was non-blinded, and the participants could receive alarms in the case of very low glucose levels. Data was extracted from the receiver using Glooko^®^ software.

### Linkage to the Danish Working Hour Database (DAD) and national health registers

The cohort is nested in DAD, which contains payroll data for all employees in the five administrative regions in Denmark (www.nfa.dk/dad) from 2007 and onwards (currently until 2020) and it is regularly being updated [17]. The database contains daily information on working hours, overtime and absence, together with information on workplace, job type, department and date of employment. Linkage to DAD via personal registration numbers implies that data from questionnaires, blood samples and technical measurements will be supplemented with detailed information about future and historical working hours potentially dating more than 15 years back in time and allowing for a precise exposure assessment. Likewise, the cohort will be linked to Danish health registers containing information about somatic and psychiatric diagnoses [19, 20] and redemption of medication [21].

### Collaboration

The data collection for at a subsample of 322 participants are part of the EU-project The Exposome Project for Health and Occupational Research (EPHOR, www.ephor-project.eu). Together with data collections in Spain, Sweden and the Netherlands, we followed the study protocol for the shift work part of EPHOR, which implied additional self-reported measures, and collection of saliva samples and light exposure.

The research team welcomes further national and international collaborations. For more information, please visit the project’s webpage (www.nfa.dk/1001nights) or contact the corresponding author.

### Statistical analyses

First, sociodemographic and health-related characteristics of the 1001 nights-cohort were compared across four groups based on their self-reported contractual work schedule (i.e. permanent day work; permanent evening work *or* 2-shift work without night work; permanent night work; 2- and 3-shift work with night work). Percentages and means with their standard deviation (SD) are presented in Table 2.

**Table 2:** Description of the study population of the 1001 nights-cohort. 994 participants responded to the questionnaire, and participants with missing data are excluded analysis by analysis

	Permanent day workers (*n* = 191, 17.8%)				Permanent evening and 2-shift work without night work (*n* = 96, 8.9%)				Permanent night workers (*n* = 74, 6.9%)				2- or 3-shift work with night work (*n* = 714, 66.4%)				Total population (*n* = 1075)			
	N	%	Mean	SD	N	%	Mean	SD	N	%	Mean	SD	N	%	Mean	SD	N	%	Mean	SD
**Sociodemographic factors**																				
Age (years)	191		44.6	10.9	96		44.8	13.2	74		46.6	11.4	714		39.5	11.7	1075		41.4	12.0
Vocational education ≥ 3 years	154	84.2			68	74.7			46	67.7			570	87.4			838	84.3		
Tenure (years)	183		8.6	8.7	91		7.6	8.2	68		8.9	7.6	652		6.8	7.4	994		7.3	7.8
**Working hours and commuting time**																				
Weekly working hours	191		36.5	3.6	96		36.6	4.2	73		34.4	4.8	712		36.5	3.6	1072		36.4	3.7
Influence on own working hours (yes)	146	80.2			71	78.0			46	67.7			466	71.6			729	73.5		
Ever had night work ^1^	72	39.3			43	47.3			68	100			624	95.7			807	81.2		
Years with night work	72		11.1	8.4	43		9.4	8.9	68		14.5	10.7	623		10.4	9.4	806		10.8	9.5
Finds night work strenuous ^2^	48	67.6			34	81.0			16	23.5			298	47.8			396	49.3		
**Health behaviors**,** somatic and mental health**																				
Smoking (daily, occasionally)	19	10.5			14	15.4			13	19.1			85	13.1			131	13.2		
Physical activity *≤* 4 h/week	91	50.3			42	46.2			37	54.4			302	46.4			472	47.6		
Poor self-reported health	11	6.1			18	19.8			12	17.7			61	9.4			102	10.3		
Self-reported diagnoses ^3^																				
High cholesterol	28	15.6			16	17.6			23	33.8			87	13.4			154	15.6		
Asthma	20	11.1			15	16.5			14	20.6			73	11.3			122	12.4		
Migraine	35	19.4			24	26.4			17	25.0			107	16.5			183	18.5		
Depression	29	16.1			19	20.9			12	17.7			108	16.6			168	17.0		
MDI-score	180		8.4	7.2	90		10.7	7.9	68		8.6	6.9	650		10.4	7.8	988		10.0	7.7
**Sleep and chronotype**																				
Poor self-reported sleep	49	27.1			42	46.2			28	41.2			225	34.6			344	34.7		
MEQ-score	181		57.2	7.6	91		53.0	7.3	67		45.2	8.9	650		52.4	7.8	989		52.8	8.3

^1^ At least 3 nights per month (night work is defined as at least 3 h between midnight and 6:00 AM)

^2^ Scale coded from 1 (not at all strenuous) to 7 (very strenuous), score 5–7 reported in table

^3^ The four most frequent diagnoses from a list of 13 diagnoses

MDI = Major Depression Inventory, MEQ = Morningness Eveningness Questionnaire, higher score equals to more morningness

Second, for the group of participants with night work, we describe the self-reported characteristics of their work schedules and preferred working hours (Table 3).

**Table 3:** Description of night shift workers of the 1001 nights-cohort

	Permanent night workers (*n* = 74)				2- or 3-shift workers with night work (*n* = 714)			
	N	%	Mean	SD	N	%	Mean	SD
Preferred number of consecutive night shifts	68		4.2	1.6	617		2.4	1.1
Works ≥ 3 consecutive night shifts on average	65	95.6			333	53.5		
Works ≥ 3 night shift per week on average	66	97.1			89	14.3		
Has the possibility of napping during shifts (Yes)	37	54.4			302	46.4		
Has a long shift (> 12 h) once a week or more	12	17.7			125	19.2		
Takes melatonin	12	17.7			90	13.8		

Third, we compared the study population of the 1001 nights-cohort with the source population based on data from DAD. For this comparison, DAD was delineated to the inclusion criteria of the 1001 nights-cohort (i.e. women, ≥ 18 of age, employed ≥ 28 h a week, no absence due to pregnancy in 2019 or maternity leave in 2020). We used 2019-data to avoid COVID-19-related anomalies in working hours. For this comparison, we extracted information on age, average weekly working hours, job groups and work schedules (Table 4).

SAS 9.4 and R were used for the statistical analyses.

**Table 4:** Comparison of the 1001 nights-cohort with the source population. The comparison is based on 2019 data, and the same register-based inclusion and exclusion criteria are applied on the two groups for this comparison

	DAD population 2019 (*n* = 94,959)				1001 nights-cohort in 2019 (*n* = 649)			
	N	%	Mean	SD	N	%	Mean	SD
Age			44.0	11.9			41.6	10.7
Job groups								
Nurses ^1^	41,457	43.7			442	68.1		
Assistant nurses ^2^	11,802	12.4			87	13.4		
Medical doctors ^3^	8,968	9.4			30	4.6		
Other care personnel ^4^	3,137	3.3			10	1.5		
Other ^5^	29,594	31.2			80	12.3		
Avg. weekly working hours^6^			32.0	4.9			32.9	5.1
Work schedule ^7^								
Permanent day	55,520	58.5			149	23.0		
Permanent night	804	0.8			17	2.6		
Shift work without night work	20,265	21.3			129	19.9		
Shift work with night work	18,370	19.3			354	54.5		

^1^ Nurses

^2^ Nursing assistants, other types of care work

^3^ Medical doctors

^4^ Midwifes, paramedics, therapists

^5^ Not covered by other categories, includes cleaning, bio analysts, psychologist, medical secretaries, etc.

^6^ Average weekly working hours across 2019 (excluding weeks with 0 working hours)

^7^ Work schedules in DAD are defined as: Permanent day: < 6.7% evening shifts and < 6.7% night shifts, permanent evening: < 6.7% day shifts and < 6.7% night shifts, permanent night: < 6.7% day shifts and < 6.7% evenings shifts

## Results

In total, 1075 female employees from Danish hospitals were enrolled in the cohort study. Among these, 18% were permanent day workers, 9% were permanent evening workers or 2-shift workers without night work, 7% were permanent night workers, and 66% were 2- or 3-shift workers with night work (Table 2). The latter group had the highest percentage of participants with a vocational education of 3 years and above (87%), whereas the lowest percentage was observed among the permanent night workers (68%). Permanent day workers and permanent night workers had a slightly longer tenure (above 8 years) than the remaining two groups, however the variation in the population is relatively large (SD > 7 years).

Permanent night workers reported fewer weekly working hours (34.4 h) than the other groups. Participants without current night work more frequently reported having influence on their own working hours compared with night working groups. Among permanent day workers and employees with permanent evening work or 2-shift work without night work, previous experience with night work was frequent (39% and 47%, respectively). Participants without night work perceived night work more strenuous than current permanent night workers and participants with 2- or 3-shift work with night work.

The highest percentage of daily/occasional smoking and physical activity ≤ 4 h per week was observed among permanent night workers. Poor self-reported health, migraine and depression was most frequent among permanent evening workers and 2-shift workers without night work. Self-reported high cholesterol and asthma were most frequent among permanent night workers. The poorest self-reported health and sleep quality were observed among permanent night workers and the combined group of permanent evening workers and 2-shift workers without night work. Assessed with the MEQ-scale, we found that permanent day workers had the highest score (i.e. early chronotype), whereas permanent night workers had the lowest score (i.e. late chronotype).

The preferred number of consecutive night shifts was 4.2 for permanent night workers and 2.4 for 2- or 3-shift workers with night work (Table 3). The majority of the permanent night workers had ≥ 3 consecutive night shifts and ≥ 3 night shifts per week, whereas participants with 2- or 3-shift work with night work had fewer consecutive night shifts and fewer night shifts per week. Napping during the night shift was possible for 54% of the permanent night workers and 46% of the participants with 2- or 3-shift work with night work.

A subsample of 649 participants of the 1001 nights-cohort were identified in DAD in 2019 (Table 4). The average weekly working hours were similar among participants and non-participants, but more participants were nurses (68% vs. 44%). As planned, the percentage of employees with shift work with night work were higher in the 1001 nights-cohort (55% vs. 19%), while there were fewer permanent day workers (23% vs. 59%) compared with the source population.

## Discussion

### Main findings and characteristics of the 1001 nights-cohort

The 1001 nights-cohort involves 1075 hospital employees, who contributed with comprehensive information obtained from an examination on the day of enrollment, self-reported information from background questionnaires and sleep diaries, continuous measurements of sleep, physical activity, light exposure, skin temperature and glucose levels via wearables, and biological markers from blood and saliva. The majority of the participants (73%) were engaged in night work, either as permanent night workers or as 2- or 3-shift workers with night work. In addition, a large proportion of current day and/or evening workers previously worked night shifts. Across all health indicators, the poorest health was observed among permanent night workers and the group of shift workers *without* night work. Compared with the source population, participants of the 1001 nights-cohort were more frequently employed as nurses and more frequently working night shifts.

### Strengths and limitations

With its high-resolution data from self-reports and technical measurements using wearables and in combination with biological data from blood and saliva samples, this cohort makes an important contribution to previous cohorts and biobanks within night work research, e.g. the Norwegian “Shiftwork and health complaints study” [22], the American “Nurses Health Study” [23], EPHOR (www.ephor-project.eu), and the Dutch Klokwerk [24]. The completion rate assessed as the number of background questionnaires and sleep diaries that were filled out was high, and only 16 participants (1.5%) actively notified the research team that they wanted to terminate the data collection before the end of the 14 days’ study period. Thus, the feasibility of the study design and data collection was high.

With the present data, acute effects of night work on musculoskeletal pain, headache, and continously measured blood glucose are investigated using a within-person design. Intermediate effects of night work on low-grade inflammation, glycated hemoglobin, and blood lipids can be investigated using a between-person design. Also, differences in other aspects of the working environment, such as psychosocial stressors and physical demands across shift types can be investigated using the present data, and data also allow comparisons of technical and self-reported measures of physical activity during work and leisure.

Future linkage to detailed register information will allow for a thorough assessment of the health of the 1001 nights-cohort compared with the source population. Register-based information about historical and future working hours and health also enables future research on both acute and long-term health effects of working hours, night-work related circadian disruption, and sleep deprivation. Furthermore, the data allow us to study the same individual under different conditions (e.g. on a day shift versus a night shift), and thus use the participants as their own control, which reduces the risk of time-invariant confounding. Importantly, as the data also makes it possible to investigate biological markers, the cohort will contribute to the knowledge of physiological mechanisms and causal relations. A subsample of the study population is contributing with data to a European cohort (EPHOR), which enables cross-country comparisons and additional analyses.

To ensure that data were collected according to the protocol, each participant received individual, oral information about the data collection, and was given written instructions on the use of all wearables that were provided. To ensure consistency throughout the data collection, project group members received a detailed manuscript and the procedures for the oral information and the measurements taken on the examination day were rehearsed together. The processes regarding data cleaning and validation of data are documented and can be accessed upon request.

We used validated scales to assess, for example, chronotype [14], depressive symptoms [15], and sleep [16]. It cannot be ruled out, however, that the detailed reporting of daily habits and use of wearables could influence behaviors, similar to a Hawthorne effect [25]. Likewise, social desirability bias may also have affected the reporting of “socially undesirable” or unhealthy behaviors [26]. Additionally, as the enrollment took place at the participants’ workplace, concerns regarding confidentiality could influence the responses, despite that all participants were informed that no information would be shared with employer or colleagues.

We cannot rule out that night work-related sleepiness and circadian disruption could influence the experience, interpretation, and reporting of other physical and psychosocial stressors as well as somatic and mental symptoms. Yet, our data are strengthened by the use of multiple data sources covering both objective and subjective measures and the possibility of combining these, which reduce the influence of common methods bias [27].

Participants were enrolled individually, and although we did not apply any health-related selection criteria, we cannot rule out that health-related selection into the cohort took place. We did not systematically collect information about reasons for participation. On an anecdotal level, some participants explained that their motivation to participate was due to struggles working night shifts. In contrast, others were motivated to participate in order to ensure that also employees, who were satisfied with night work were represented in the data. Data from population-based health surveys show that participants have a lower all-cause mortality than non-participants [28, 29]. Yet, in a cohort study on occupationally active adults aged 46 and 52 years at the time of recruitment, no substantial difference in sociodemographic factors (e.g. sex, occupation, and contractual hours) between participants and non-participants was observed [30].

In the present study, the overall “healthy worker-effect” is likely to operate along with a “healthy shift worker-effect” [31]. The direction of this selection is difficult to determine. Yet, permanent day workers seemed to report better health on most health indicators than shift workers. Among the shift workers, employees with 2- or 3-shift work with night work reported better health and sleep.

The participants’ exposure to night work (or other working hour characteristics) was not manipulated during the study period. Instead, the study relied on observational data reflecting participants’ existing work schedules. Additionally, participants were encouraged to work, sleep, eat, and exercise as they used to. Therefore, the cohort contributes with knowledge about how free-living individuals behave and adapt to their environment, and the effects hereof. Although the underlying physiological mechanisms must be assumed to be the same regardless of whether the exposure is determined by the researcher or the participant, causal interpretation and generalization should be done with caution, and sufficient attention should be paid to selection mechanisms and confounders.

As this is an occupational cohort comprising employees from the secondary health care sector, participants are, on the one hand, relatively homogenous. On the other hand, the data collection took place at 26 hospitals across the country, and the sample therefore consists of urban as well as more rural/suburban areas. The cohort also covers a variety of job groups within the health care sector with nurses and other care personnel being the most frequent. Solely female employees were eligible for inclusion. This methodological decision was taken to optimize the use of resources, as males are underrepresented at regional workplaces and even more in the job groups that were most prone to participate in the data collection (22% males among permanent day workers and 19% among other groups) [17]. Thus, analyses do not need stratification by sex but can be run in the full sample, which increases statistical power. Obviously, the disadvantage is that results from this cohort do not necessarily apply to male employees and to other job groups, and generalization depends on findings from cohorts and field studies including men and other sectors [32, 33].

## Conclusion

Results from the present cohort can substantially advance research-based knowledge about short- and long-term effects of circadian disruption on human health and will be able to deliver important input for updating existing guidelines on healthy scheduling of working hours. The abundance of data can be used for studies on, for example, sleep-related outcomes, patterns in diet and physical activity, and the interplay between circadian disruption and psychosocial exposures, individual preferences, chronotype, and mental and physical health.
